# Supplementary material for: Multiple repeat regions within mouse DUX recruit chromatin regulators to facilitate an embryonic gene expression program
Source: bioRxiv. 2023 Jun 8:2023.03.29.534786. Originally published 2023 Apr 1. Preprint. [Version 2] doi: 10.1101/2023.03.29.534786 (PMC10081216; doi:10.1101/2023.03.29.534786)
Supplement: Supplement 3 — Supplementary Figure 1: DUX orthologs do not contain a repeat structure similar to mouse DUX. a) Human DUX4 aligned to itself. b) Rat DUX4 aligned to itself. c) Bovine DUXC aligned to itself. d) Table with a pairwise comparison of e-values from a BLAST-based amino acid similarity comparing mouse DUX C-terminal repeats, the human DUX4 C-terminus, the rat DUX4 C-terminus, and the bovine DUXC C-terminus. Parameters are PAM250 matrix and gap costs of existence: 15 and extension: 3. *No significant similarities found (>0.05). e) Experimental design for expression of mCherry-tagged DUX domain constructs and flow cytometry of GFP% | mCherry tag expression. f) Western blot for mCherry tag after 18hr expression of C1, C12, C123, C1234, C12345. Predicted protein sizes are 59 kDa (C1), 69 kDa (C12), 79 kDa (C123), 89 kDa (C1234), 99 kDa (C12345), 48 kDa (∆C12345+14aa), and 100 kDa (FL). Supplementary Figure 2: DUX homeodomains are required for transcriptional activity. a) (Left) Schematic of constructs used for flow cytometry: mCherry-tagged full-length DUX (FL), ΔC12345+14aa, C12345Δ14aa, C1+14aa, C12+14aa, C123+14aa, C1234+14aa, C5+14aa, C45+14aa, C345+14aa, C2345+14aa, ΔHDs, ΔHD1, ΔHD2, and Dux−/− alone. (Right) Flow cytometry for MERVL::GFP reporter given mCherry expression in Dux−/− mESCs following 18hr expression of indicated constructs. *p-value < 0.05, student’s t-test. (n=3 biological replicates). b) (Left) Schematic of additional constructs used for flow cytometry: mCherry-tagged full-length DUX (FL), C3+C5+14aa, C3+C5Δ14aa, C124+14aa. (Right) Flow cytometry for MERVL::GFP reporter given mCherry expression in Dux−/− mESCs following 18hr expression of indicated constructs. *p-value < 0.05, student’s t-test. (n=3 biological replicates) c) Schematic of C1C3 chimera DUX constructs for amino acid cut offs for C1C3a, C3C3b, C1C3c, C3C1a, C3C1b, and C3C1c aligned to the 5 C-terminal repeats d) Heat map of RNA-seq at DUX target genes (n=456) (21) from 18hr expression of DUX [file NIHPP2023.03.29.534786v2-supplement-3.pdf]

Supplementary Figure 1

bioRxiv preprint doi: https://doi.org/10.1101/2023.03.29.534786; this version posted June 8, 2023. The copyright holder for this preprint (which was not certified by peer review) is the author/funder, who has granted bioRxiv a license to display the preprint in perpetuity. It is made available under aCC-BY 4.0 International license.

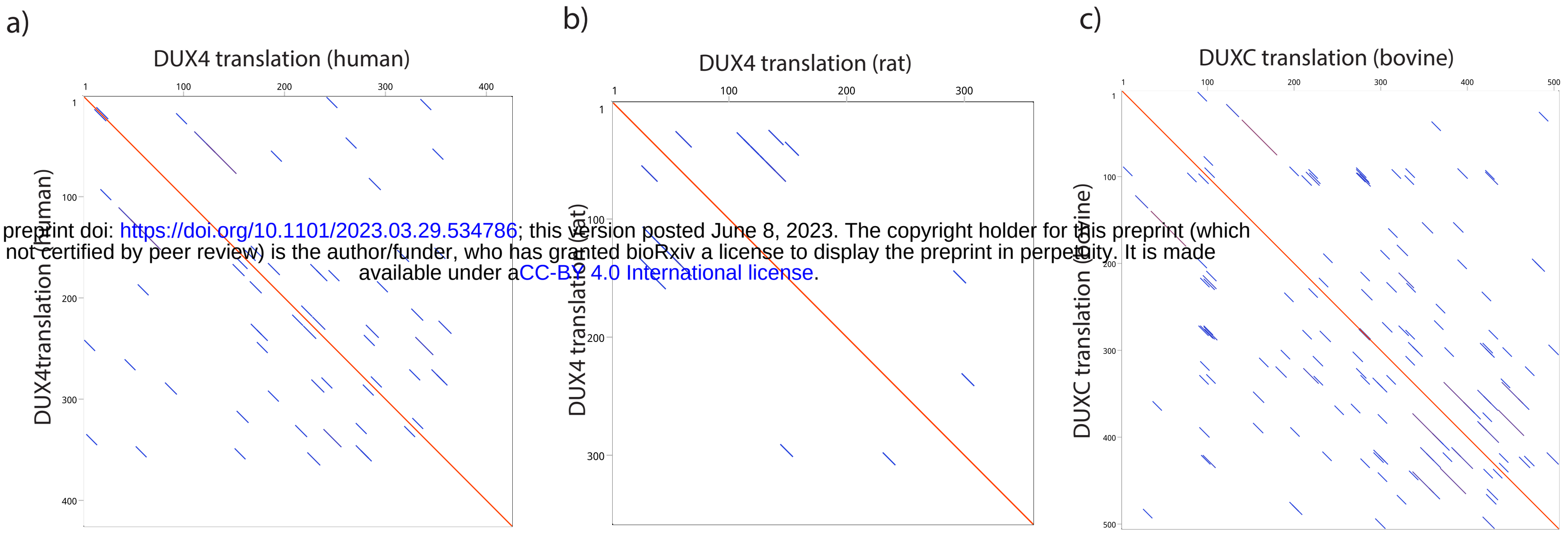

d)

	C-term repeat 1	C-term repeat 2	C-term repeat 3	C-term repeat 4	C-term repeat 5	DUX4 C-term	DUXC C-term	Dux4 C-term
**C-term repeat 1**		3e-24	4e-36	7e-22	2e-25	*	0.024	0.0009
**C-term repeat 2**	3e-24		1e-34	1e-41	2e-23	0.030	*	*
**C-term repeat 3**	4e-36	1e-34		9e-31	7e-42	*	*	*
**C-term repeat 4**	7e-22	1e-41	9e-31		1e-09	*	*	0.002
**C-term repeat 5**	2e-25	2e-23	7e-42	1e-09		0.022	0.016	0.035
**DUX4 C-term (H)**	*	0.030	*	*	0.022		1e-06	*
**DUXC C-term (B)**	0.024	*	*	*	0.016	1e-06		*
**DUX4 C-term (R)**	0.0009	*	*	0.002	0.035	*	*	

* No significant similarities found (>0.05)

e) Schematic for experimental design for all flow cytometry in Dux domain constructs

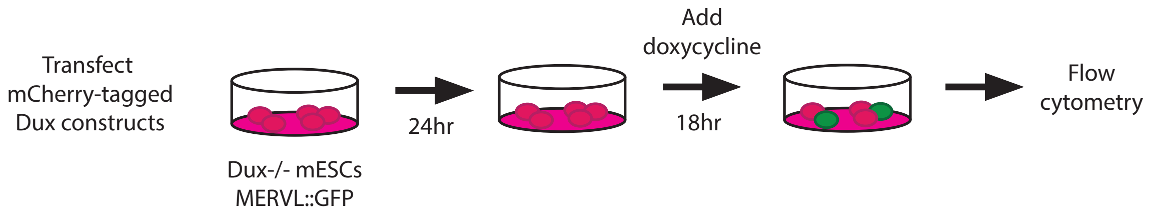

f) Western blot for mCherry-tagged Dux domain constructs

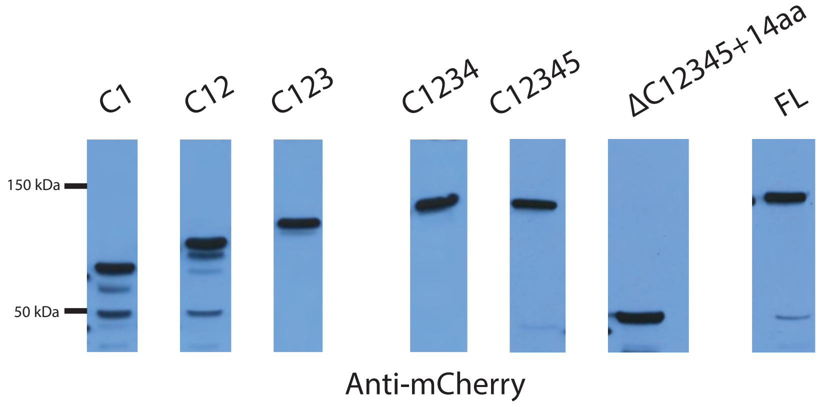

Supplementary Figure 2

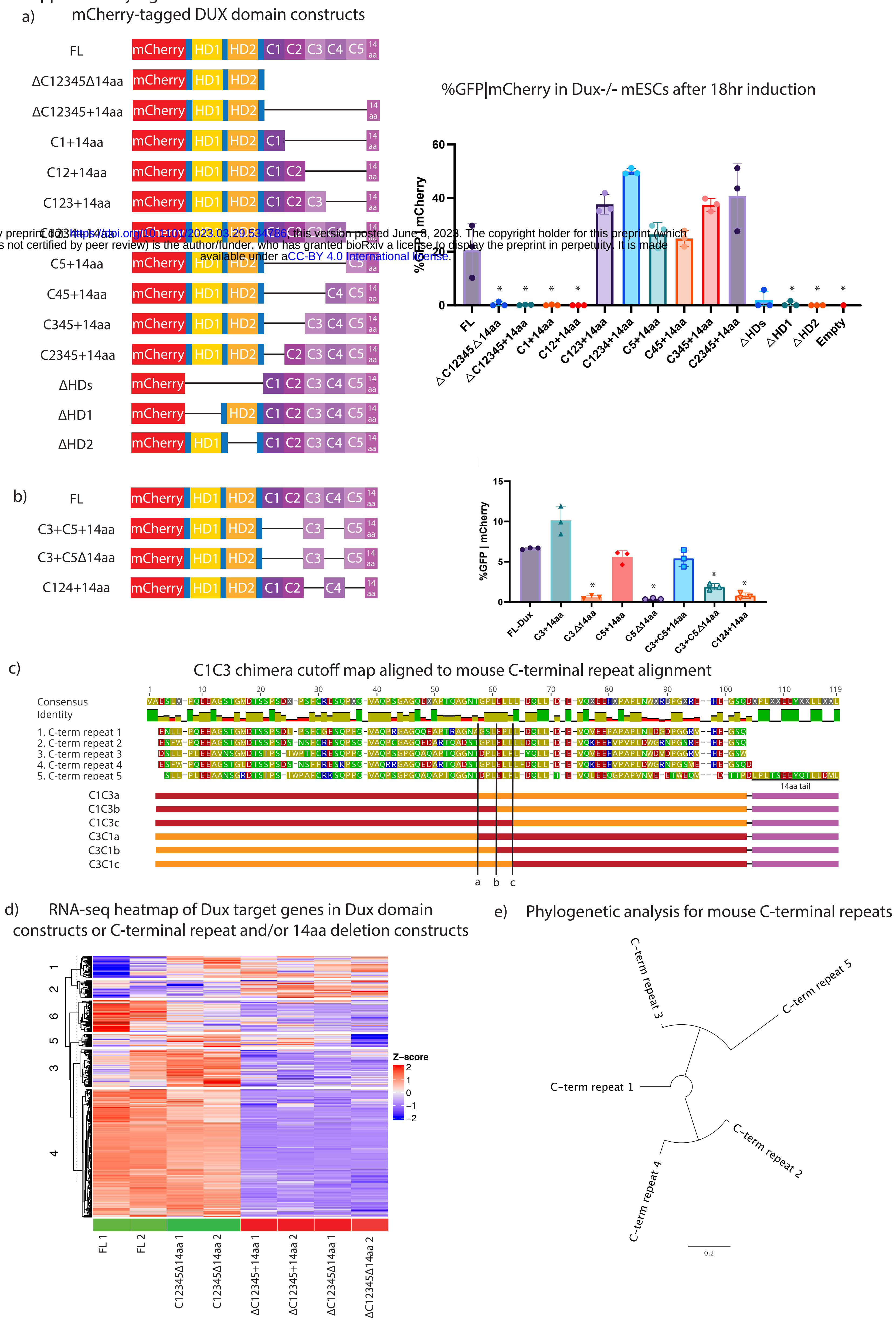

bioRxiv preprint doi: https://doi.org/10.1101/2023.03.29.534786; this version posted June 8, 2023. The copyright holder for this preprint (which was not certified by peer review) is the author/funder, who has granted bioRxiv a license to display the preprint in perpetuity. It is made available under aCC-BY 4.0 International license.

Supplementary Figure 3

a) Profile plot of DUX ChIP-seq vs. mCherry-tagged DUX CUT&Tag at DUX binding sites

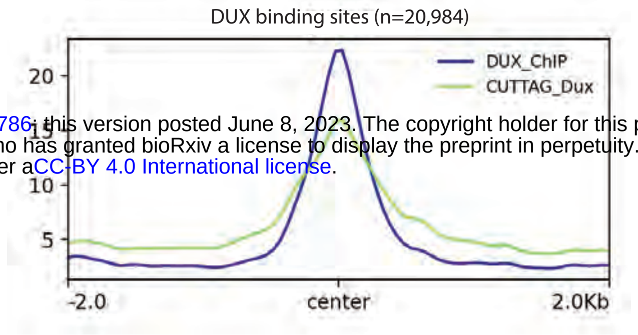

bioRxiv preprint doi: https://doi.org/10.1101/2023.03.29.534786; this version posted June 8, 2023. The copyright holder for this preprint (which was not certified by peer review) is the author/funder, who has granted bioRxiv a license to display the preprint in perpetuity. It is made available under aCC-BY 4.0 International license.

b) IGV screenshots of mESC H3K9ac and DUX-expressing cells H3K9ac at DUX target genes

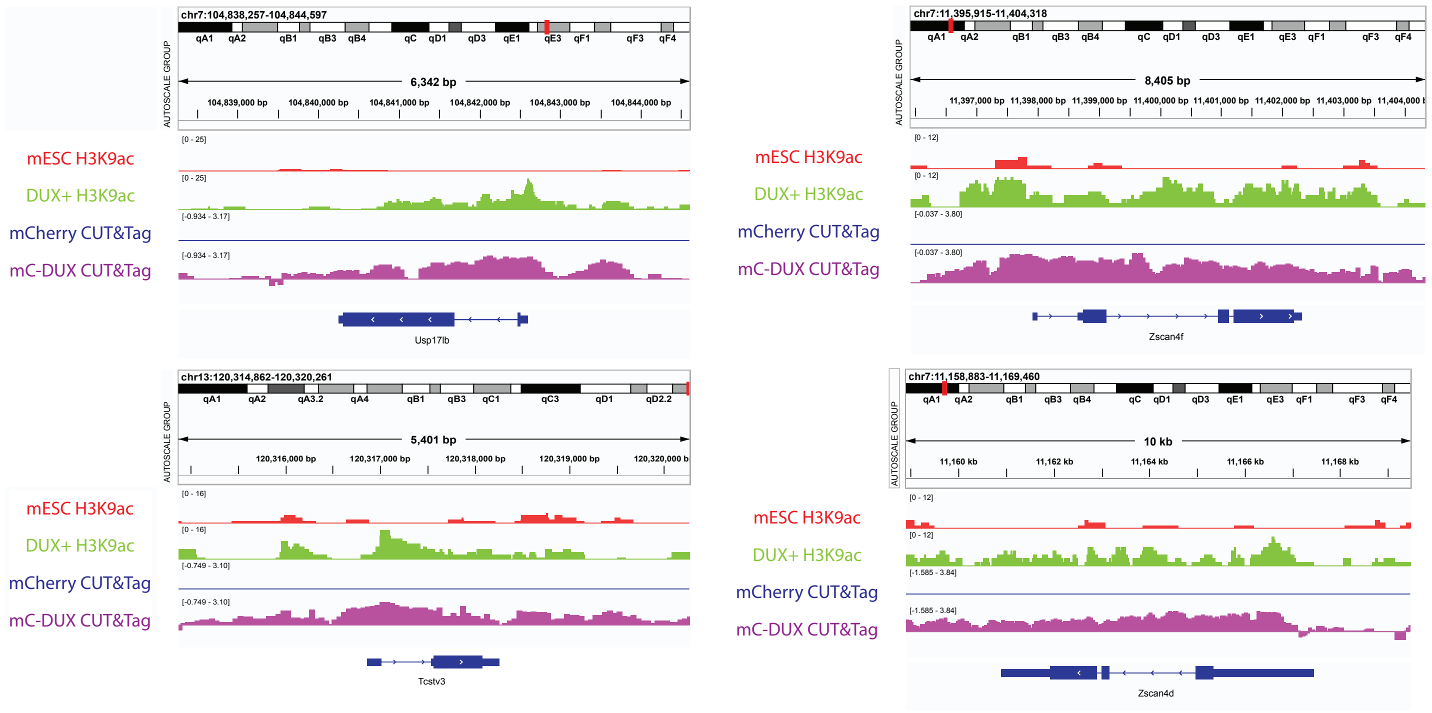

Supplementary Figure 4

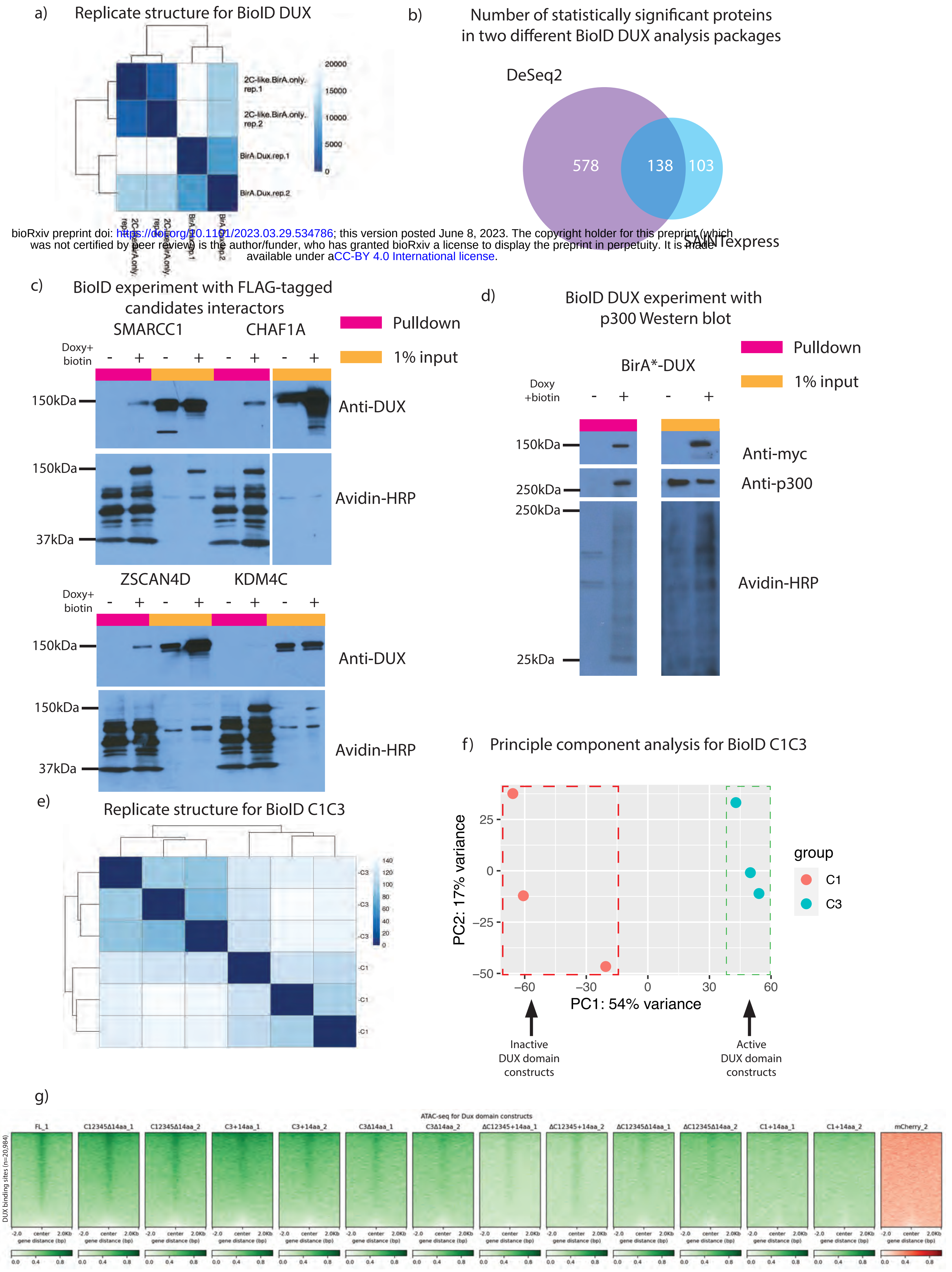

bioRxiv preprint doi: https://doi.org/10.1101/2023.03.29.534786; this version posted June 8, 2023. The copyright holder for this preprint (which was not certified by peer review) is the author/funder, who has granted bioRxiv a license to display the preprint in perpetuity. It is made available under aCC-BY 4.0 International license.
